# Evaluating Short-Term Outcomes of Tunneled and Non-Tunneled Central Venous Catheters in Hemodialysis

**DOI:** 10.3390/jcm13133664

**Published:** 2024-06-23

**Authors:** Niccolò Morisi, Martina Montani, Edwidge Ntouba Ehode, Grazia Maria Virzì, Salvatore Perrone, Vittoria Malaguti, Marco Ferrarini, Gabriele Donati

**Affiliations:** 1 Surgical, Medical, Dental and Morphological Sciences Department (CHIMOMO), University of Modena and Reggio Emilia, 41126 Modena, Italy; 2 Nephrology, Dialysis and Kidney Transplant Unit, Azienda Ospedaliero-Universitaria di Modena, 41121 Modena, Italy; 3 IRRIV—International Renal Research Institute Vicenza Foundation, 36100 Vicenza, Italy; 4 Department of Nephrology, Dialysis and Transplantation, San Bortolo Hospital, 36100 Vicenza, Italy

**Keywords:** hemodialysis, central venous catheter, tunneled CVC, non-tunneled CVC, catheter complications, acute kidney failure, short-term prognosis, catheter-related infections

## Abstract

**Background**: The necessity of using central venous catheters (CVCs) in hemodialysis, coupled with their associated complications, remains a critical concern in nephrology. This study aims to compare the short-term prognosis of tunneled (T-CVC) and non-tunneled (NT-CVC) CVCs in acute hemodialysis patients, specifically focusing on infection rates, malpositioning, and lumen thrombosis within the first three weeks post-insertion. **Methods**: A retrospective analysis was conducted on 176 CVCs placed between January and December 2023 at the Policlinico di Modena and the Ospedale Civile di Baggiovara. Patient demographics, CHA2DS2-VASc scores, and comorbid conditions were recorded at the time of catheter placement. Outcomes assessed included catheter-related infections, malpositioning, and lumen thrombosis. Statistical analyses, including Chi-square tests, Fisher’s exact tests, and Kaplan–Meier survival analysis, were performed to evaluate differences between T-CVCs and NT-CVCs. **Results**: The sample comprised 43% females with a mean age of 69.3 years (SD 13.9) and a mean CHADS-VASC score of 3.72 (SD 1.4). Hypertension (90%) was the most prevalent comorbidity. Of the 176 CVCs, 127 were T-CVCs and 49 were NT-CVCs. Infection rates were 3.15% for T-CVCs and 8.16% for NT-CVCs (*p* = 0.07). Malpositioning occurred in 0.79% of T-CVCs and 4.08% of NT-CVCs (*p* = 0.47). There was one case of lumen thrombosis in the NT-CVC group. Kaplan–Meier analysis indicated a significant divergence in infection-related catheter survival favoring T-CVCs after ten days (*p* = 0.034). **Conclusions**: While non-tunneled CVCs do not significantly alter short-term prognosis compared to tunneled CVCs, the latter show a better infection-related survival rate beyond ten days. Therefore, primary insertion of T-CVCs may be preferable when resources and clinical conditions permit, although NT-CVCs remain a viable option when immediate T-CVC insertion is challenging.

## 1. Introduction

In their seminal 1999 article, Schwab and Beathard brought to light a crucial clinical issue in hemodialysis: the necessity of using central venous catheters (CVCs) and the challenges that come with them [1]. Despite extensive efforts globally to increase the use of arteriovenous fistulas, the prevalence of CVCs has continued to rise. Between 1996 and 2007, the use of catheters among hemodialysis patients increased by 1.5- to 3-fold across many countries, including among non-diabetic patients aged 18 to 70 years. Additionally, a significant proportion of patients newly diagnosed with end-stage renal disease (ESRD), ranging from 58% to 73%, relied on catheters for initiating hemodialysis [2]. The persistent use of hemodialysis CVCs highlights their indispensable role in contemporary nephrology [3]. The dilemma pointed out by Schwab and Beathard remains pertinent: nephrologists are challenged by the complications associated with catheters but must rely on them due to their critical utility in hemodialysis. Long-term catheterization is often associated with significant issues such as thrombosis, vascular stenosis, and infections [4,5]. To mitigate these problems, the industry has developed new CVC designs incorporating technical innovations to enhance patient safety and catheter performance. Additionally, improvements in CVC design have also aimed to make the insertion procedure easier and safer.

CVCs are mainly categorized into tunneled (T-CVC) and non-tunneled (NT-CVC) types [6]. Traditionally, T-CVCs have been designed for long-term use to reduce infection risks, whereas NT-CVCs have been intended for short-term access due to their higher complication rates. The KDIGO guidelines suggest changing or removing NT-CVCs within two weeks, specifically recommending the removal of non-tunneled jugular CVCs within this period [7]. However, this conventional distinction is increasingly being questioned, especially in the context of acute patients. For instance, a review by Matthew J. Oliver observed that the risk of infection between the two catheter types might not be as clear-cut as previously thought. The study found that the incidence of infection was higher in tunneled catheters compared to non-tunneled ones, likely due to the short duration of use [8]. Indeed, the risk of bacteremia is well connected to the permanence of NT-CVCs [9]. Despite KDIGO recommendations, some centers manage patients for months to years with NT-CVC for management needs or in low-resource environments. One study reported a bacteremia rate of only 1.2 per 1000 catheter days with long-term use [10]. This center uses dry gauze and povidone-iodine to dress catheter exit sites. Both povidone-iodine and mupirocin ointments with dry gauze dressings have been shown to significantly reduce the risk of bacteremia from acute dialysis catheters in randomized controlled trials [11].

The choice between tunneled and non-tunneled CVCs remains a topic of considerable debate among healthcare providers. Previous studies have noted differences in infection rates, mechanical complications, and patient survival between these catheter types. This study aims to evaluate whether placing a tunneled CVC (T-CVC) immediately is more advantageous than initially using a non-tunneled CVC (NT-CVC) in acute patients. By comparing the short-term outcomes of these two catheter types, our primary endpoints include catheter-related infections, mechanical complications, and patient survival rates. Understanding these factors is essential for optimizing hemodialysis treatment and improving patient outcomes.

## 2. Materials and Methods

This retrospective study included patients who had had CVCs placed between 1 January 2023 and 31 December 2023 at the Policlinico di Modena and the Ospedale Civile di Baggiovara. The study evaluated the short-term outcomes of tunneled (T-CVC) versus non-tunneled (NT-CVC) central venous catheters in hemodialysis patients within the first three weeks of placement. There is no established protocol for selecting between T-CVC and NT-CVC. The decision to place an NT-CVC or T-CVC was made on a case-by-case basis, taking into account both clinical and organizational factors. The followed protocol for CVC insertion was utilized in all our patients [12,13]. The choice of insertion site, whether the internal jugular vein or the femoral vein, depends on the patient’s clinical condition [14]. Ultrasound guidance is used to visualize and select the optimal insertion site. All patients receive antibiotic prophylaxis with ceftazidime or ciprofloxacin for penicillin allergy. Creating a sterile field involves rigorous hand washing followed by donning sterile gloves, mask, gown and cap. The insertion site is disinfected with chlorhexidine. For the placement of an NT-CVC (Duo-Split, SEDA s.p.a.), local anesthesia with mepivacaine was performed. The guide needle is inserted into the vein under ultrasound guidance, followed by the insertion of a guidewire through the needle. After removing the needle, the tract is dilated with a dilator, and the catheter is inserted over the guidewire. The guidewire is then removed, and the correct position of the catheter is verified by aspirating blood and flushing with saline. The catheter is secured to the skin with sutures or adhesive and covered with a sterile dressing. The placement of a T-CVC (Palindrome, Medtronic Italia s.p.a.) involves a similar process, with additional steps to create a subcutaneous tunnel. Local anesthesia is administered to both the insertion site and the tunnel exit site. After inserting the guide needle into the vein and passing the guidewire through it, a subcutaneous tunnel is created from the insertion site to the exit site using a tunneller. The catheter is then passed through this tunnel. The tract is dilated with a dilator, and the catheter is inserted over the guidewire. Following the removal of the guidewire, the catheter’s position is confirmed by aspirating blood. The catheter is secured and covered with a sterile dressing. At the end, verification of the catheter’s position for the jugular site is typically performed through a chest X-ray. Subsequent management of the CVC involves the use of heparin as locking solution and the use of unfractionated heparin or low-molecular-weight heparin during the session [15].

We included all patients who received either a T-CVC or NT-CVC for hemodialysis during the specified period at the two hospitals. Exclusion criteria were applied to patients who had a catheter placed for reasons other than hemodialysis and those who had incomplete medical records.

The data were collected retrospectively from patient medical records and included demographic information (age, sex, underlying conditions), type of catheter placed (T-CVC or NT-CVC), and specific outcomes within the first three weeks post-placement. Additionally, an assessment of patients’ clinical characteristics was performed at the time of catheter placement. This included the calculation of the CHA2DS2-VASc score (congestive heart failure, hypertension, age > 75 years, diabetes, prior stroke/transient ischemic attack, vascular disease, age 65–74 years, female sex) [16], and documentation of any acute (<30 days) or chronic (>30 days) infectious conditions and oncological disease.

The primary outcomes evaluated in this study were catheter-related infections, malpositioning of the catheter, and lumen thrombosis. These outcomes were assessed within the first three weeks following catheter placement to determine the immediate risks and complications associated with each type of CVC. Following local protocol, all NT-CVCs were removed after 21 days, and T-CVCs were positioned for patients requiring hemodialysis. Descriptive statistics were used to summarize the baseline characteristics of the patient population, including the CHA2DS2-VASc scores, the presence of acute or chronic infections, and oncological conditions.

Continuous variables were presented as means and standard deviations (SDs) or medians and interquartile ranges (IQRs) as appropriate, while categorical variables were presented as frequencies and percentages. To compare the incidence of primary outcomes (catheter-related infections, malpositioning, and lumen thrombosis) between the T-CVC and NT-CVC groups, the chi-squared test was employed. For categorical variables with expected frequencies less than five, Fisher’s exact test was used. Relative risk (RR) and 95% confidence intervals (CIs) were calculated to compare the risk of each outcome between the two catheter types. Kaplan–Meier survival curves were constructed to compare the time to occurrence of each outcome between the T-CVC and NT-CVC groups, with the log-rank test used to assess the statistical significance of differences between the survival curves. All statistical analyses were performed using SPSS (Statistical Package for the Social Sciences), with a *p*-value of less than 0.05 considered statistically significant.

## 3. Results

In our sample, 43% of patients were female with a mean age of 69.3 years (standard deviation (SD) 13.9). The mean CHA2DS2-VASC score was 3.72 (SD 1.4). The most common medical condition was hypertension (90%), followed by vascular disease (61%), heart disease (55%), and diabetes (49%) [17,18]. Approximately one-third of patients had chronic infectious problems (35%) mainly viral conditions such as HBV, HCV, and HIV. A similar proportion of patients had active or anamnestic cancer problems (31%), mainly affecting colon, lung, and breast. See Table 1 for more information.

A total of 176 catheters were inserted, 127 of them were T-CVCs and 49 were NT-CVCs. In this study, we evaluated the presence of adverse events during the first 21 days of permanence of CVCs. The total number of CVCs affected by adverse events was 26 and 19 of them were NT-CVC. Among the NT-CVCs, 11 were placed in the femoral site and 8 in the jugular site. No significant differences were observed between the femoral and jugular NT-CVCs regarding infection rates, with a total of four events (two for the femoral NT-CVCs and two for the jugular NT-CVCs, *p* = 0.574). One case of malposition was observed for the jugular NT-CVCs, while one case of thrombosis was observed for the femoral NT-CVCs. Concerning catheter-related infections, we observed four infections in each group, giving an incidence of 3.12% for T-CVCs and 8.16% for NT-CVCs (*p* = 0.07). When examining the relative risk (RR) of catheter-related infections, T-CVCs compared to NT-CVCs were at 0.39, indicating a lower risk associated with T-CVCs. All the infections detected needed the removal of the CVC; in five cases, patients needed to be hospitalized. None of the infection events affected patients with immunosuppressive therapy. Similarly, malpositioning occurred in one case for T-CVCs and one case for NT-CVCs (*p* = 0.47), giving an incidence of 0.79% and 2.04%, respectively. In addition, a single case of luminal thrombosis was observed in the NT-CVC group, giving an incidence of 2.04%. For a summary see Table 2. In addition, in 10 cases (4 T-CVCs), catheter removal was prompted by recovery of renal function, allowing hemodialysis therapy to be discontinued. The mean time of restoring kidney function was 12 days. However, due to the infrequent occurrence of malpositioning and lumen thrombosis, a reliable calculation of RR for this outcome was not feasible. There were no statistical differences among the groups according to gender or comorbidity. There was no correlation between infection and age of patients (*p* = 0.56) or CHA2DS2-VASC score (*p* = 0.58). Malposition problems demonstrated a correlation with age (*p* = 0.019) and the CHA2DS2-VASC score (*p* = 0.047). The occurrence of thrombosis events was not analyzed due to the limited sample size. The results are summarized in Table 2.

**Figure 1:**
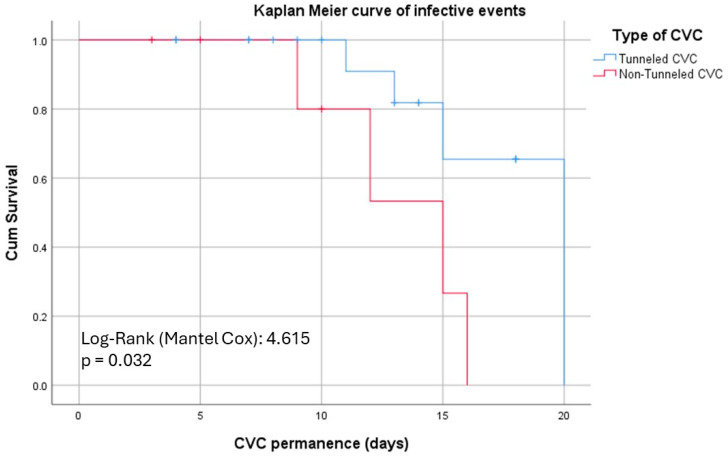
Kaplan–Meier curves comparing the time to catheter removal due to infectious events between T-CVCs (blue line) and NT-CVCs (red line). The analysis shows a significant divergence between the two groups starting around day 10, with a *p*-value of 0.034, indicating a statistically significant difference in infection-related catheter survival in favor of T-CVCs. CVC, central venous catheter; T-CVC, tunneled CVC; NT-CVC, non-tunneled CVC.

A Kaplan–Meier analysis (see Figure 1) was performed to assess infectious events of T-CVCs and NTCVCs associated with them, revealing a significant distinction between the two curves around the tenth day (*p* = 0.032).

## 4. Discussion

The primary cause of complications associated with CVCs in acute settings is infection, while issues such as malposition or catheter lumen thrombosis are relatively rare. Our findings align with the current literature [19,20,21], highlighting the ongoing evaluation of strategies to mitigate infection risks [22,23,24]. Despite the extensive use of CVCs and the innovations in their design aimed at improving safety and performance, the challenge of managing infections remains significant.

In our study, the substantial number of infectious complications enabled a thorough statistical comparison of infection events between NT-CVCs and T-CVCs. Despite the difference in sample size between the two groups, the differences in infection rates within the first three weeks post-catheter placement were not statistically significant. This observation is corroborated by existing studies [25,26] which similarly report that short-term use of NT-CVCs does not result in significantly higher infection rates compared to T-CVCs.

However, Kaplan–Meier analysis provides a more nuanced understanding of infection risks over time. Initially, within the first ten days, there is no significant difference in infection rates between T-CVCs and NT-CVCs. Beyond this period, the curves begin to diverge significantly, with T-CVCs showing a lower incidence of infections. This supports a superior survival rate for tunneled catheters in terms of remaining infection-free. This finding aligns with the KDIGO guidelines, which recommend the replacement of NT-CVCs within two weeks to reduce infection risks.

Further supporting our data, several studies indicate that functional recovery in acute patients typically occurs after approximately two weeks [27,28]. This trend was also evident in our patient cohort. Consequently, many patients will require a second interventional procedure to replace NT-CVCs with T-CVCs, despite the potential for discontinuation of dialysis soon. This necessity for catheter replacement underscores the importance of initial catheter choice and the potential benefits of opting for a T-CVC when clinically feasible.

The initial placement of a T-CVC can pose challenges, such as the patient’s clinical condition and the operator’s expertise with the procedure [29,30], In these scenarios, the use of NT-CVCs is justified and, as our data confirm, does not result in a higher rate of short-term complications. However, in the absence of these barriers, the immediate use of a T-CVC is preferable. This approach not only aligns with infection prevention strategies but also reduces the need for subsequent catheter replacement, thereby minimizing additional interventions and associated risks [21,31].

Moreover, the economic and logistical implications of multiple catheter placements should be considered. Each additional procedure carries risks of complications, increased healthcare costs, and patient discomfort 31. By opting for T-CVCs initially in suitable patients, healthcare providers can enhance patient outcomes and potentially reduce the overall burden on healthcare resources.

This study has several limitations that should be acknowledged. Firstly, given the retrospective design of this study, the blinding of the investigators or patients was not feasible. As the data had already been collected, the investigators were aware of the catheter types during data analysis, which could introduce bias in the assessment of outcomes. Secondly, the study was conducted in two specific hospitals, which may limit the generalizability of the findings to other settings with different patient populations and healthcare practices. Additionally, the relatively small sample size, particularly for certain complications like lumen thrombosis, may affect the statistical power and the ability to detect significant differences between the groups. A potential bias may be the presence of immunosuppressive therapy, particularly in patients with active oncological problems. However, the low number of cases and the absence of infectious complications in the short term preclude this possibility. It cannot be ruled out that this condition may manifest itself over a longer follow-up period. Finally, the study did not account for potential confounding factors such as variations in operator expertise, catheter insertion techniques, and post-insertion care protocols, which could influence the outcomes. Future prospective studies with larger sample sizes and more standardized protocols are needed to validate these findings and provide more definitive recommendations.

## 5. Conclusions

The findings of this study suggest that the primary insertion of T-CVCs could be a preferable approach in acute hemodialysis patients when resources and expertise are available. T-CVCs demonstrated a lower risk of infection beyond the initial ten-day period compared to NT-CVCs, supporting their use for better long-term outcomes. However, in scenarios where the primary insertion of T-CVCs is challenging due to clinical conditions or limited operator expertise, the use of NT-CVCs remains a viable and justified option. Importantly, our data indicate that NT-CVCs do not adversely affect short-term prognosis, providing flexibility in catheter choice without compromising immediate patient safety. This evidence underscores the need for a tailored approach, ensuring that each patient’s circumstances and the healthcare facility’s capabilities are considered to optimize care and outcomes.

## Figures and Tables

**Table 1:** General characteristics of population and divided by type of central venous catheter (CVC). No significant differences were observed between groups. n, numbers; T-CVC, tunneled CVC; NT-CVC, non-tunneled CVC; SD, standard deviation; %, percentage.

	General (n = 176)	T-CVC (n = 127)	NT-CVC (n = 49)	*p*-Value
Male; n (%)	101 (57)	56 (44)	19 (39)	0.66
Age; mean (SD)	69.3 (13.9)	68.3 (14.8)	71.9 (10.9)	0.12
Diabetes; n (%)	87 (49)	62 (49)	25 (51)	0.7
Hypertension; n (%)	158 (89)	119 (93)	39 (79)	0.06
CHF history; n (%)	97 (55)	72 (56)	25 (51)	0.59
Stroke/TIA history; n (%)	18 (10)	11 (9)	7 (14)	0.31
Vascular pathology; n (%)	107 (61)	80 (63)	27 (55)	0.42
Oncology pathology; n (%)	54 (31)	41 (32)	13 (27)	0.57
Chronic infection; n (%)	61 (35)	47 (37)	14 (29)	0.38
CHA2DS2-VASc score; mean (SD)	3.72 (1.4)	3.67 (1.4)	3.85 (1.47)	0.45
Antibiotics therapy; n (%)	11 (6)	8 (6)	3 (7)	0.82
Immunosuppressive therapy; n (%)	4 (2)	3 (2)	1 (2)	0.95

**Table 2:** Adverse events are divided by type of CVC. CVC, central venous catheter; n, numbers; %, percentage; T-CVC, tunneled CVC; NT-CVC, non-tunneled CVC; nd, not determined.

	T-CVC (n = 127)	NT-CVC (n = 49)	*p*-Value
Infection; n (%)	4 (3.15)	4 (8.16)	0.07
Malposition; n (%)	1 (0.79)	1 (2.04)	0.47
Thrombosis; n (%)	0	1 (2.04)	Nd

## Data Availability

Data are unavailable due to privacy restrictions.
